# Epidemiology of Poisoning Cases at Lumbini Medical College, Nepal: A 5‐Year Retrospective Study

**DOI:** 10.1002/hsr2.71000

**Published:** 2025-07-10

**Authors:** Samata Nepal, Alok Atreya, Prakriti Regmi, Prasun Shrestha, Rishav Babu Shrestha, Laxmi Prasad Sapkota, Ritesh G. Menezes, Himal Ghimire, Suraj Dhakal, Shikha Pahari

**Affiliations:** ^1^ Department of Community Medicine Lumbini Medical College Palpa Nepal; ^2^ Department of Forensic Medicine Lumbini Medical College Palpa Nepal; ^3^ Lumbini Medical College Palpa Nepal; ^4^ Forensic Medicine Divison, Department of Pathology, College of Medicine Imam Abdulrahman Bin Faisal University Dammam Saudi Arabia; ^5^ Department of Internal Medicine Lumbini Medical College Palpa Nepal; ^6^ Department of Emergency Medicine Lumbini Medical College Palpa Nepal; ^7^ Department of Nursing Lumbini Medical College Palpa Nepal

**Keywords:** epidemiology, mental health, Nepal, pesticides, poisoning, suicide prevention

## Abstract

**Background and Aims:**

Poisoning is a major global public health problem, disproportionately burdensome in low‐resource healthcare settings. The objective of this study was to gain epidemiological insights into poisoning cases at Lumbini Medical College and Teaching Hospital over a period of 5 years.

**Methods:**

A retrospective cross‐sectional analysis was performed of hospital records and 402 poisoning cases admitted to Lumbini Medical College between January 2019 and May 2024 were analyzed descriptively using SPSS V.27.0. The study meticulously documented a wide range of data, such as patient demographics, types of poisons, contexts for poisoning, clinical presentation, and medical outcomes.

**Results:**

Of 402 cases, females predominated (61.2%, *n* = 246), with a median age of 26.5 years (IQR: 18.2–39.2). Pesticides, particularly organophosphates, were the leading agent (57.7%, *n* = 232), with self‐harm as the primary reason (70.9%, *n* = 285), often linked to family conflict. Poisoning peaked in the evening, notably among females aged 25–44 years (42.3%, *n* = 170). Mortality was low (0.2%, *n* = 1), with 68.7% of patients receiving some form of psychiatric consultation.

**Conclusions:**

Poisoning primarily involved young, married females using organophosphorus pesticides for self‐harm, often due to family conflict and mental health issues, with most receiving psychiatric consultation.

## 1 Introduction

Poisoning is a significant and preventable public health concern worldwide, including Nepal, where it represents one of the primary causes of emergency department (ED) visits and hospitalization [1, 2]. According to the World Health Organization (WHO), approximately 0.3 million people die annually from various poisoning agents, with pesticides being the leading cause [1]. Poisoning, both intentionally and accidentally, can lead to serious medical complications. Due to advances in technological and social developments that have made most drugs and chemical substances more widely available in society, the true incidence of poisoning events could be underestimated because of underreporting [3].

Household chemical agents and prescribed drugs are the most common poisoning agents in the developed world, but agrochemicals are the most common poisoning agents in developing countries [4, 5].

According to the national data for the 2017 and 2018 period, 11,136 suicide cases due to poisoning were reported to the police, of which more than 90% were attributed to pesticides [6]. According to WHO data, suicide mortality in Nepal in 2019 was reported to be 8.99 per 100,000 people, which is 0.17 more than that in 2018, indicating a worsening situation [7]. The Global Burden of Disease (GBD) Study 2019 reported a significant reduction in unintentional poisoning mortality across South Asia, with the age‐standardized death rate decreasing by 55.88% from 0.87 (95% CI 0.67–1.01) per 100,000 in 1990 to 0.41 (95% CI 0.34–0.47) per 100,000 in 2019 [8]. However, Nepal exhibited a unique trend, with an increase in unintentional poisoning incidence (IRR 0.97, 95% CI 0.94–1.00), contrasting with reductions in Bangladesh (−61.8% in DALYs) and India (−60.65% in age‐standardized death rate) [8]. This regional variability underscores the need for localized studies, such as ours, to elucidate specific patterns and drivers of poisoning in Nepal, particularly in understudied areas like Lumbini Province.

The objective of this study was to investigate the epidemiology of poisoning cases at Lumbini Medical College (LMC) over a period of 5 years.

## 2 Methods

A retrospective cross‐sectional study was conducted using hospital records at Lumbini Medical College and Teaching Hospital, a tertiary care facility in Palpa district, Lumbini Province, Nepal. Serving a wide region, including all of Palpa district and parts of neighboring districts such as Gulmi, Arghakhanchi, and Syangja, LMC's catchment area covers a population exceeding 1 million. According to the LMC annual report, out‐patient department (OPD) visits increased from 49,818 in the Nepali fiscal year 2079/80 (approximately July 16, 2022, to July 15, 2023) to 83,512 in 2080/81 (approximately July 16, 2023, to July 15, 2024), with in patient department (IPD) admissions increasing from 4517 to 7158 over the same periods. This suggests a core population of 300,000–500,000, based on estimated utilization rates of 2–3 OPD visits per person and 50–100 IPD admissions per 1000 people annually. The Nepali fiscal year, spanning mid‐July to mid‐July (e.g., Shrawan 1 to Ashad 31), differs from the Gregorian calendar.

Ethical approval was obtained from the Institutional Research Committee at LMC (IRC‐LMC‐06/Q‐23). Due to the retrospective nature, patient consent to participate was waived per IRC‐LMC approval.

The study included all poisoning cases admitted to the ED, including those referred from other wards, between January 1, 2019, and May 15, 2024. Eligible cases involved patients with a confirmed diagnosis of poisoning based on clinical assessment, encompassing both intentional and unintentional exposures.

Patients with incomplete medical records were excluded from the study.

Relevant medical records were obtained from the Medical Record Department (MRD) by searching files and registers using the hospital number of patients admitted due to poisoning. A standard proforma was designed for data collection, assigning a unique identifier to each case. Demographic details (age, sex/gender, place of residence, and marital status) and poisoning details (date and time, substance category/toxic agent, route of exposure, reason for poisoning, diagnosis, duration of hospital stay, and medical outcome) were recorded, with personal identifiers omitted. Substance categories included therapeutic drugs, pesticides (insecticides, rodenticides, and insect repellents with insecticidal properties), alcohol (limited to ethanol, with no cases of toxic alcohols identified), chemicals, food, deliriants (*Datura* species, known for hallucinogenic properties), and unknown agents. Toxic agents were classified as unknown when the poison could not be identified or was suspected but unconfirmed, with asymptomatic cases assessed separately using clinical severity scales. Reasons for poisoning were classified as intentional (self‐harm) or unintentional (accidental exposure) based on patient history, with an unknown category for cases lacking sufficient information, aligning with standard toxicological reporting practices.

Statistical analyses were performed using Microsoft Excel 2007 and SPSS V.27.0 for Windows. Results were reported as means with standard deviations (SDs) or medians with interquartile ranges (IQRs) for continuous variables, chosen for their suitability in describing retrospective demographic and clinical data. Due to non‐normal distribution of vital signs data (Shapiro–Wilk test, *p* < 0.001), Mann–Whitney *U* tests were used to compare vital parameters between accidental and self‐harm groups, with a *p*‐value < 0.05 considered statistically significant. A descriptive analysis of the reasons for poisoning was conducted based on the case history. Age groups were standardized as 0–14, 15–24, 25–44, 45–64, and 65+ to align with WHO classifications and the study's demographic distribution.

## 3 Results

The study included 402 patients admitted with poisoning between January 2019 and May 2024. Females represented 61.2% (*n* = 246) of cases, whereas males accounted for 38.8% (*n* = 156). The age range of the patients was from 1 to 80 years, with a median age of 26.5 years (IQR: 18.2–39.2).

**Figure 1:**
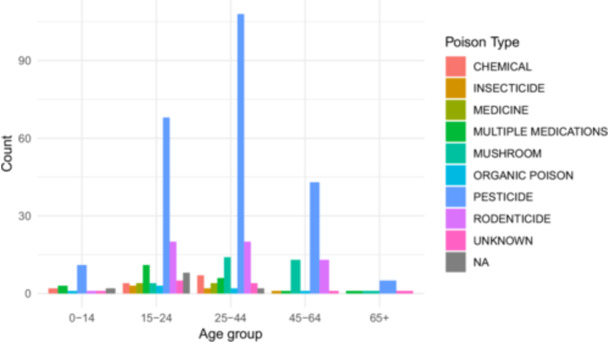
Substance category involved by age group.

Among the 402 patients analyzed, 25% were aged less than 21 years and 75% were aged less than 39.2 years (Figure 1). Most of the patients were married (65.7%, *n* = 264), followed by unmarried individuals (32.6%, *n* = 131), and a small portion were widowed (1.7%, *n* = 7) (Table 1).

**Table 1:** Demographic characteristics of the patients (*n* = 402).

Variables		Frequency *N* (%)
Age (years)	0–14	21 (5.2)
	15–24	130 (32.3)
	25–44	170 (42.3)
	45–64	73 (18.2)
	65+	8 (2.0)
Sex	Male	156 (38.8)
	Female	246 (61.2)
Marital status	Unmarried	131 (32.6)
	Married	264 (65.7)
	Widowed	7 (1.7)

**Figure 2:**
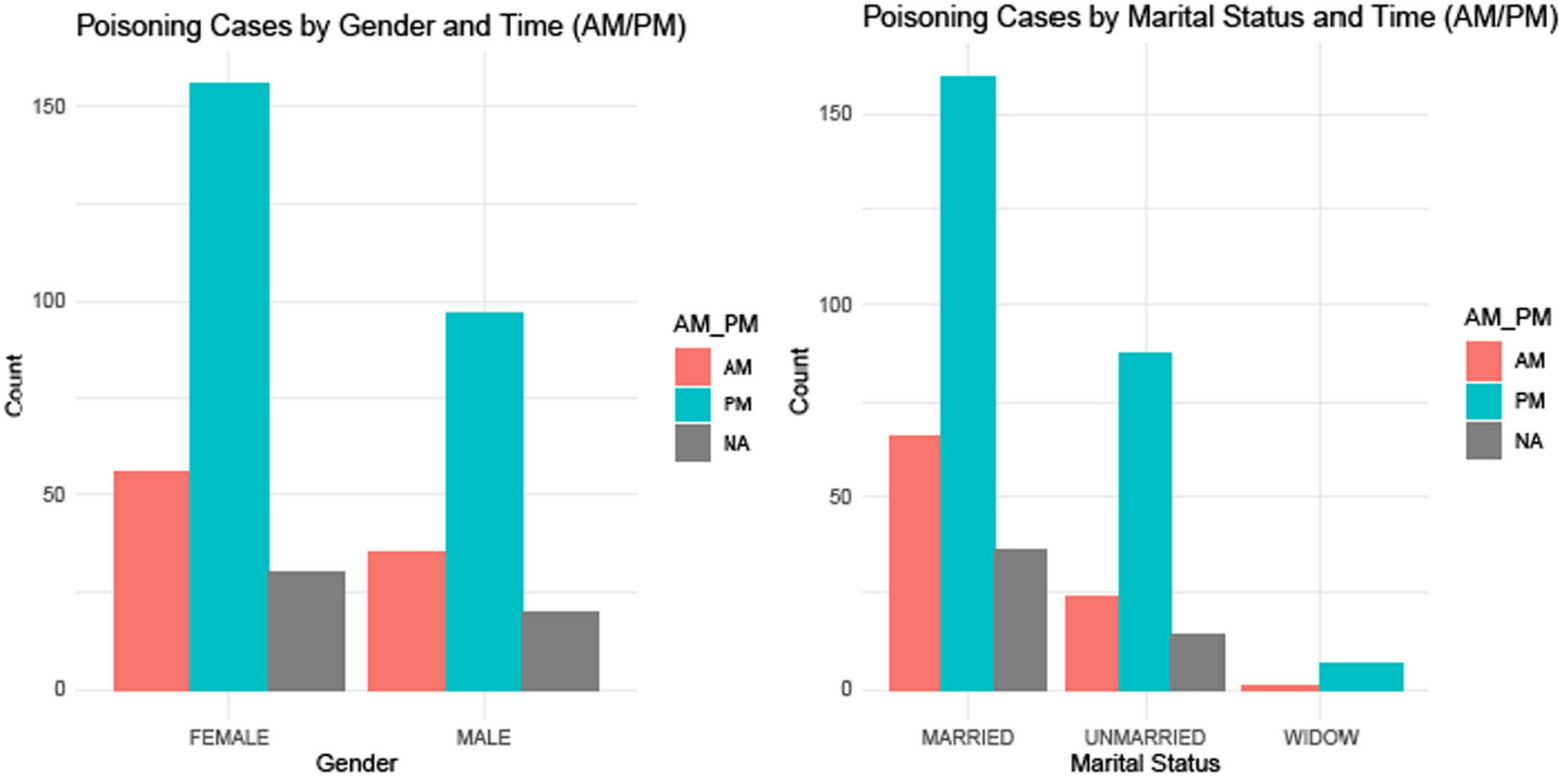
Poisoning incidence by gender (left) and marital status (right).

**Figure 3:**
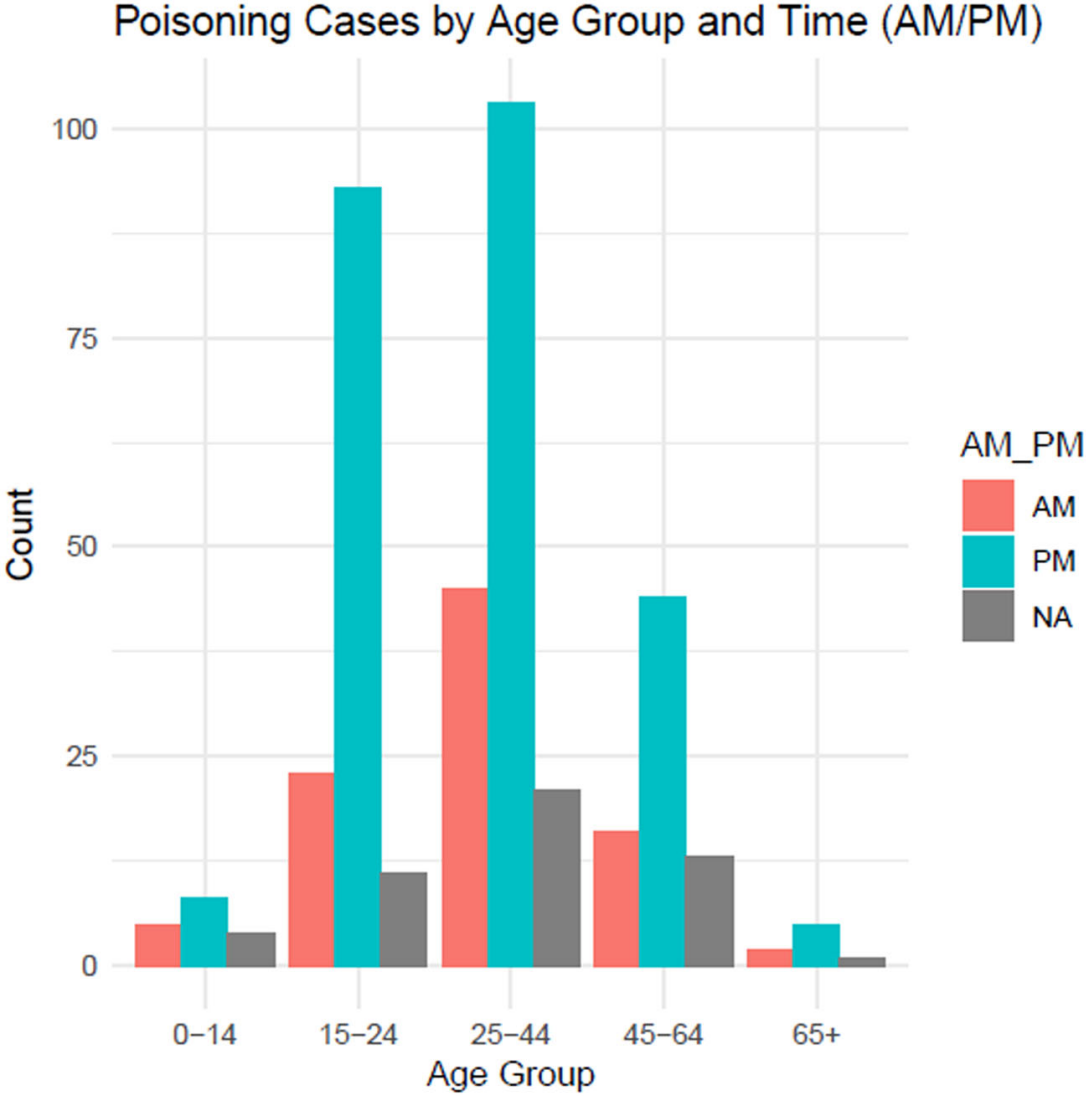
Time of poisoning incident by age group.

As shown in Figure 2, the majority of patients who ingested poison did so during the evening hours. Poisoning cases peaked in the evening, notably among females (61.2%, *n* = 246), and most married patients also took poison during this time. Figure 3 shows that the highest number of poisoning incidents occurred in the late afternoon, with many cases involving individuals aged 25–44 years (42.3%, *n* = 170).

### 3.1 Types of Poison

Pesticides, including insecticides, rodenticides, and insect repellents, were responsible for 76.4% (*n* = 307) of poisoning cases. Among the pesticides, organophosphates were the most common, accounting for 57.7% (*n* = 232) of the total cases, followed by rodenticide poisoning (14.4%, *n* = 58). Accidental mushroom poisoning accounted for 8% (*n* = 32) of the total cases. Five percent (*n* = 20) of the total patients were poisoned by therapeutic drugs. Other poison types included multiple medications, insect repellents, deliriants, and unspecified substances, with a smaller distribution across cases (Table 2).

**Table 2:** Substance categories involved in poisoning.

Substance category	Frequency	Percentage
Insecticide	232	57.7114
Rodenticide	58	14.4278
Mushroom	32	7.9601
Single medicine	20	4.9751
Multiple medications	11	2.7363
Insect repellent	11	2.7363
Unknown	9	2.2388
*Datura*	7	1.7412
Unknown pesticide	6	1.4925
Others	16	3.9800
Total	402	100.0

### 3.2 History and Clinical Examination

The route of exposure was ingestion in 99.5% (*n* = 400) of cases, with the remaining cases comprising one case exposed through ingestion, injection, and inhalation (0.2%, *n* = 1) and one case exposed through inunction (0.2%, *n* = 1). The reason for poisoning was intentional (self‐harm) in 70.9% (*n* = 285) of cases, accidental in 28.9% (*n* = 116), and unknown in one case. In terms of consciousness upon admission, 90.8% (*n* = 365) of the patients were conscious, 8.5% (*n* = 34) were unconscious, and 0.2% (*n* = 1) were drowsy. The vital parameters of the patient at presentation to the ED are shown as depicted in Table 3, reported as means with SD. Significant differences between accidental and self‐harm poisoning cases were observed in heart rate (*p* = 0.03), respiratory rate (*p* = 0.04), and temperature (*p* = 0.01), with accidental cases showing higher mean values for heart rate (96.47 vs. 90.81 bpm) and respiratory rate (22.92 vs. 21.42/min), and a slightly higher temperature (97.85°F vs. 97.71°F). No significant differences were found in systolic (*p* = 0.89) or diastolic blood pressure (*p* = 0.74).

**Table 3:** Vitals at presentation.

Vital parameters	Accident	Self‐harm	*p* value
Systolic BP (mmHg)	114.36 (16.50)	114.59 (15.72)	0.89
Diastolic BP (mmHg)	77.18 (10.15)	77.14 (10.55)	0.74
Heart rate (bpm)	96.47 (20.79)	90.81 (13.70)	0.03
Respiratory rate (/min)	22.92 (5.77)	21.42 (3.15)	0.04
Temperature (°F)	97.85 (0.56)	97.71 (0.56)	0.01

### 3.3 Provided Consultations

Only 1% (*n* = 4) of the patients received medicolegal consultation, whereas 68.7% (*n* = 276) received psychiatric consultation. For 99% (*n* = 398) of the cases, medicolegal consultations were not mentioned, and psychiatric consultation was not noted for 31.3% (*n* = 126).

### 3.4 Reasons for Poisoning

There were 116 cases of accidental poisoning and 285 cases of self‐harm. Alcohol intoxication accounted for 7.5% (*n* = 30) of total cases, with 5.0% (*n* = 20) linked to intentional self‐harm among adults and 2.5% (*n* = 10) unintentional, including one case of a 5‐year‐old boy who accidentally ingested ethanol. The major underlying cause of deliberate self‐harm caused by poisoning was found to be familial conflict, mental health issues, financial hardship, relationship problems, and academic stress, as depicted in Table 4.

**Table 4:** Reasons for poisoning as per the provided history.

Reason for poisoning	Possible underlying factor	Frequency *N* (%)
Intentional	Family conflict	120 (29.9)
	Mental health issues	27 (6.7)
	Economic stress	17 (4.2)
	Relationship problems	8 (2.0)
	Emotional disturbance	14 (3.5)
	Alcohol intoxication	20 (5.0)
	Academic stress	9 (2.2)
	Not mentioned	70 (17.4)

### 3.5 Outcome

The hospital stay ranged from 1 to 15 days, with a mean duration of 4.44 ± 2.287 days. A majority of patients (90.8%, *n* = 365) did not follow up postdischarge, whereas 9.2% (*n* = 37) attended follow‐up sessions. In terms of medical outcomes, 400 patients were discharged, one patient (a 19‐year‐old unmarried female, intentional organophosphorus insecticide poisoning via ingestion, with a history of mental health issues) was referred to a specialized facility after 10 days of hospitalization for advanced critical care due to persistent complications, and one patient (a 59‐year‐old married male, intentional organophosphorus insecticide poisoning via ingestion under the influence of alcohol for self‐harm) died after 5 days of admission.

## 4 Discussion

### 4.1 Demographic Characteristics

This study revealed a distinctive demographic profile of poisoning cases. The median age was 26.5 years, with a female predominance (61.2%), younger than the mean ages reported at Scheer Memorial Adventist Hospital (37.12 ± 14.07 years for organophosphate cases, 27.41 ± 9.79 years for non‐organophosphate cases) [9]. Studies on poisoning from other Asian countries depict a similar picture in which female patients are more at risk and adolescents and young adults constitute a vulnerable age group [4, 10].

Although married individuals accounted for the majority of poisoning cases (65.7%), this percentage was marginally lower than that reported in a record‐based study from southern India, which reported that 74.8% of the cases were among married people [11]. This finding on age and gender suggests that social and familial factors may contribute to poisoning, warranting future investigation into these potential risk factors.

### 4.2 Poisoning Patterns

Although organophosphate dominates poisoning cases at LMC (57.7%, *n* = 232), reflecting their accessibility in Nepal's agrarian context, the GBD Study 2019 indicates a broader range of causative agents for unintentional poisoning in South Asia, including carbon monoxide and other chemicals [8]. The GBD data show a 63.8% reduction in age‐standardized death rates from “other means” (e.g., pesticides, drugs) between 1990 and 2019, contrasting with a smaller 33.1% decline for carbon monoxide‐related deaths [8]. This suggests that although unintentional pesticide poisoning may be decreasing regionally, intentional pesticide use, prevalent in our study, remains a critical public health challenge in Nepal, potentially driven by psychosocial factors rather than accidental exposure.

Ingestion emerged as the most predominant route of poisoning, accounting for 99.5% of the cases. This method of exposure aligns with the findings of a study of poisoning cases visiting the ED of Kathmandu Medical College Teaching Hospital, where ingestion accounted for 86.57% of the cases [12].

### 4.3 Temporal and Contextual Factors

Family conflicts emerged as the primary reason for intentional poisoning, followed by mental health issues. This pattern mirrors findings from Nepal Medical College and Teaching Hospital, Kathmandu [2].

On the other hand, patients considered suicide attempts to manipulate the environment or to seek attention as per a study conducted among patients visiting psychiatric OPDs at Shree Birendra Hospital, Kathmandu, Nepal [13].

Academic stress contributes to self‐harm in students. However, the pattern of poisoning is different in medical and nursing students, who have abused prescription drugs and medications more than pesticides. One of the reasons might be that they know the properties and treatment of the drugs and that these medications are more easily accessible to them than are pesticides [14, 15].

The high prevalence of intentional poisoning among young adults may be due to low job opportunities and economic achievement, which lead to the loss of hope [10, 16].

In the present study, a characteristic time pattern of poisoning incidents was also observed, with a large proportion of exposure in the evening hours. This evening peak, particularly among females and married individuals aged 25–44 years is in line with a Nepali study conducted in Chitwan which also observed high number of cases (43.7%) in the evening hours (median hour: 17:22, mode: 22:00 h) [26]. GBD Study's long‐term trend analysis shows a consistent decline in unintentional poisoning mortality across South Asia from 1990 to 2019 (−55.88% in age‐standardized death rate) [8]. Nepal, however, bucks this trend with a slight increase in unintentional poisoning incidence (IRR 0.97, 95% CI 0.94–1.00) [8], suggesting that local factors—such as evening stress accumulation or pesticide availability—may sustain poisoning rates despite regional improvements. This temporal clustering warrants further investigation into diurnal risk factors specific to Nepal. Similar to studies on human mortality that show circadian rhythms in death rates [17], the results of the present study indicate that poisoning incidents may also exhibit nonrandom temporal distributions. The timing of poisoning may be affected by several factors, such as the psychological stress that accumulates throughout the day, decreased social support or supervision during evening hours, the culmination of interpersonal conflicts or emotional stress, and potential associations with work or family stress patterns [17]. Further prospective studies are needed to examine temporal clustering in poisoning.

### 4.4 Clinical Management and Outcomes

The treatment of the poisoning cases was determined according to the substance category, time of recognition of poisoning, age of the patient, and poison‐related factors. On a case‐by‐case basis, both pharmacological and nonpharmacological care were provided. Gastric lavage was performed in 19.9% of the patients, similar to the 17% reported in an Ethiopian study [3], and charcoal was used in only 16.2% of the total patients. The most frequent pharmacological treatment was atropine, the antidote for organophosphate poisoning. This finding was consistent with other Nepalese studies [6, 18].

Organophosphorus compound poisoning causes profound bradycardia and hypotension. The peradeniya organophosphorus poisoning (POP) scale, which is used to assess clinical severity and determine prognosis in poisoned patients, utilizes six clinical parameters, including the respiratory rate and heart rate [19].

Supportive care, including fluid administration and decontamination, is crucial in managing poisoning complications. These results suggest that most treatments are supportive or symptomatic. This may be because of the limited ability of standardized diagnostic laboratory tests to ascertain the precise chemical agent so that the clinician can administer a specific antidote [20]. Similarly, a study in India reported that 80.2% of the cases had undergone gastric lavage and that 40% of the poisoning cases were treated with IV fluids [21].

The study demonstrated an exceptionally low mortality rate of 0.2%, which was significantly lower than that reported in an Iranian study (2.3%) [22] and a Nepalese study (5%) [23].

In an autopsy‐based study from Palpa, Nepal, there were only 9 cases of poisoning of 184 cases, accounting for 4.9% of the total autopsies conducted in the mortuary of the District Hospital, of which 3 were male and 6 were female [24]. A hospital‐based study of poisoning from eastern Nepal reported 17.6% mortality out of 85 cases of poisoning in 2 years [25]. A descriptive epidemiological study from 2015 on insecticide poisoning in central Nepal reported 439 cases of acute pesticide poisoning during a 9‐month study, 3.8% of which died [26]. Similarly, a study in Ethiopia reported 1.5% mortality among poisoning cases [27]. The lower mortality in the case of pesticide poisoning may be due to early transport to the hospital, decontamination by gastric lavage and charcoal, the availability of specific antidotes, and the management of complications with intensive care support [28].

### 4.5 Health Impact Beyond Mortality

Although our study reports a low mortality rate (0.2%), the broader health impact of poisoning, including disability, remains unquantified. The GBD Study 2019 estimates that unintentional poisoning in South Asia resulted in 34,053.8 disability‐adjusted life‐years (DALYs) per 100,000 in 2019, with a 5.9% increase in years lived with disability (YLDs) [8]. At LMC, the predominance of organophosphates (57.7%, *n* = 232), aligning with national trends where OPs account for 39.6%–65% of poisonings [29], and the high intentionality (70.9%) suggest a significant hidden burden. Nationally, pesticide suicides rose 13‐fold from 1980 to 2019, with methyl parathion and dichlorvos historically prominent despite bans (e.g., 2006, 2019), indicating persistent exposure risks [29]. In Lumbini Province, studies report similar OP dominance (e.g., 50/138 cases, 2014–2017) with low mortality (0.7%), mirroring our findings and hinting at a survivor cohort at risk of chronic morbidity [29].

Given the 68.7% psychiatric consultation rate and low follow‐up (9.2%) at LMC, survivors—particularly young women (61.2%)—may face prolonged psychological and neurological impairments, such as depression or OP‐induced neuropathy, not captured by mortality statistics. Utyasheva et al. [29] note that 87%–97% of poisonings are intentional, yet mortality ranges from 3% to 18.7%, suggesting many survive with sequelae [29]. Estimating DALYs and YLDs could reveal this burden, especially as regulatory efforts (e.g., 2019 bans on dichlorvos, aluminum phosphide) may reduce deaths but not morbidity if smuggling or misidentification persists. Enhanced forensic toxicology and hospital surveillance, as recommended by Utyasheva et al. [29], are critical to quantify these impacts and inform post‐discharge care strategies.

### 4.6 Consultations and Follow‐Up

The majority of the patients in the present study received psychiatric consultation, and a few received additional medicolegal consultation. The primary reason for poisoning in the present study was suicidality as a result of stress. Among the numerous factors contributing to stress, the most common are family conflict, broken relationships, financial hardship, and academic pressure [28, 30, 31]. Female youth are vulnerable, as they are not able to cope with stressful life situations [28, 32]. Mental illness is considered a social taboo in the country; therefore, reluctance is observed among patients seeking medical care [33]. Unlike people with physical illnesses, which attract immediate medical attention, mentally ill people rarely seek medical care [33]. The majority of patients, once cured of poisoning and free of signs and symptoms, seek discharge without completing psychiatric counseling sessions. They rarely follow up at the psychiatry department for the completion of counseling. Without treating the root cause of suicide, mere treatment of the poisoning leaves them vulnerable to future suicides. A history of suicide, depression, and other mental illness is a major risk factor for future suicide, especially within 5 years of a previous suicide attempt [34].

### 4.7 Public Health Implications

This study reveals critical public health insights that demand targeted interventions.

- Young adults, particularly females aged 25–44 years, represent the most at‐risk population for poisoning incidents. The high prevalence of poisoning among married individuals suggests the need for targeted mental health and family support programs.
- Organophosphate pesticides account for more than 57% of poisoning cases, indicating an urgent need for strict regulation of pesticide storage and distribution. The GBD Study 2019 attributes South Asia's declining unintentional poisoning mortality to improved healthcare access and poison control measures [8]. In Nepal, where organophosphates drive both intentional (70.9%) and unintentional (28.9%) poisoning, adopting similar strategies—such as establishing functional poison control centers in Lumbini Province and enhancing toxicovigilance—could reduce exposure risks. Coupled with our findings on low follow‐up (9.2%), integrating psychiatric care with poison control services could address the dual burden of acute poisoning and chronic mental health outcomes, aligning with regional best practices.
- Family conflicts, mental health issues, and economic stress emerge as primary drivers of intentional poisoning. Although 68.7% of patients received psychiatric consultation, only 9.2% followed up after discharge. Therefore, enhancing mental health support systems, the destigmatization of mental health treatment, comprehensive follow‐up care for suicide attempt survivors, and community‐based stress management programs are recommended.
- The concentration of poisoning incidents during evening hours suggests a need for increased social support during potentially high‐stress periods, crisis intervention strategies targeted at specific times of day, and improved evening mental health support services.
- There is a growing concern of youth suicide in Nepal, especially in the Palpa district, where the number of cases is increasing [35, 36].
- The development of a comprehensive suicide prevention program focused on the early identification of at‐risk individuals is therefore recommended. It is also necessary to establish mental health resources that are easily accessible. There should be targeted interventions for youths experiencing stress, in addition to economic and social support mechanisms.
- There should be an improved protocol for medicolegal and psychiatric consultation processes and comprehensive patient follow‐up. There should be enhanced toxicological diagnostic capabilities. Integrated care models addressing both physical and mental health need to be created.

These implications underscore the complex nature of poisoning as a public health issue, emphasizing the need for a multifaceted approach involving healthcare, social services, and community‐based interventions.

### 4.8 Limitations and Future Research Directions

There are limitations identified in the present study. First, this was a single‐center, retrospective designed study, which limits the generalizability of the findings. This study relied on hospital records; however, there might have been underreporting of poisoning cases.

Future research can include multicenter, prospective studies, studies on comprehensive mental health intervention programs and community‐based poison prevention strategies, and detailed psychiatric follow‐up studies.

## 5 Conclusions

This study highlights poisoning as a significant public health issue in Nepal, predominantly affecting young, married females (61.2%, *n* = 246) due to organophosphate insecticides (57.7%, *n* = 232), with family conflicts and mental health issues as key drivers of intentional poisoning. The low mortality rate (0.2%) reflects effective medical interventions, yet the high incidence of poisoning, particularly among young adults, highlights the need for comprehensive prevention strategies. These include mental health support, pesticide regulation, and community‐based interventions—specifically, implementing pesticide safety training and expanding psychiatric follow‐up clinics—to address both exposure risks and underlying psychosocial factors.

## Author Contributions

**Samata Nepal:** conceptualization, data curation, formal analysis, writing – original draft, writing – review and editing, methodology. **Alok Atreya:** writing – original draft, writing – review and editing, data curation, investigation. **Prakriti Regmi:** investigation, methodology, writing – original draft. **Prasun Shrestha:** methodology, investigation, writing – original draft. **Rishav Babu Shrestha:** methodology, investigation, writing – original draft. **Laxmi Prasad Sapkota:** methodology, formal analysis, writing – review and editing. **Ritesh G. Menezes:** project administration, supervision, writing – review and editing, resources. **Himal Ghimire:** visualization, validation, writing – review and editing. **Suraj Dhakal:** validation, visualization, writing – review and editing. **Shikha Pahari:** project administration, resources, writing – review and editing.

## Conflicts of Interest

The authors declare no conflicts of interest.

## 1 Transparency Statement

The lead author, Samata Nepal, affirms that this manuscript is an honest, accurate, and transparent account of the study being reported; that no important aspects of the study have been omitted; and that any discrepancies from the study as planned (and, if relevant, registered) have been explained.

## Data Availability

The datasets generated during and/or analyzed during the current study will be made freely available if requested to the corresponding author.
